# First Trimester Prenatal Care Initiation Among Hispanic Women Along the U.S.-Mexico Border

**DOI:** 10.1007/s10995-017-2374-0

**Published:** 2017-12-01

**Authors:** Katherine Selchau, Maricela Babuca, Kara Bower, Yara Castro, Eugenie Coakley, Araceli Flores, Jonah O. Garcia, Maria Lourdes F. Reyes, Yvonne Rojas, Jason Rubin, Deanne Samuels, Laura Shattuck

**Affiliations:** 1 California Border Healthy Start+ Project, Project Concern International (PCI) U.S. & Border Programs, 4305 University Ave, Suite 345, San Diego, CA 92105 USA; 2 Santa Cruz County Healthy Start, Mariposa Community Health Center, 1852 N. Mastick Way, Nogales, AZ 85621 USA; 3 Ben Archer Health Center, Welcome Baby Program, 1600 Thorpe Rd, Las Cruces, NM 88012 USA; 4 44 Farnsworth Street, Boston, MA 02210 USA; 5 BCFS Health and Human Services, Healthy Start Laredo, 7019 Village Blvd., Suite 205, Laredo, TX 78041 USA; 6 La Clinica De Familia, Healthy Start Program, 575 South Alameda Blvd., Las Cruces, NM 88005 USA; 7 PCI, California Border Healthy Start+, 4305 University Ave, Suite 345, San Diego, CA 92105 USA; 8 PCI, 5151 Murphy Canyon Road, Suite 320, San Diego, CA 92123 USA; 9 P. O. Box 814358, Hollywood, FL 33081 USA

**Keywords:** Pregnancy trimester, first, U.S.-Mexico border, Hispanic Americans, Pregnant women, Prenatal care

## Abstract

*Background* First trimester prenatal care (FTPNC) is associated with improved birth outcomes. U.S.-Mexico border Hispanic women have lower FTPNC than non-border or non-Hispanic women. This study aimed to identify (1) what demographic, knowledge and care-seeking factors influence FTPNC among Hispanic women in border counties served by five Healthy Start sites, and (2) what FTPNC barriers may be unique to this target population. Healthy Starts work to eliminate disparities in perinatal health in areas with high poverty and poor birth outcomes. *Methods* 403 Hispanic women of reproductive age in border communities of California, Arizona, New Mexico and Texas were surveyed on knowledge and behaviors related to prenatal care (PNC) and basic demographic information. Chi square analyses and logistic regressions were used to identify important relationships. *Results* Chi square analyses revealed that primiparous women were significantly less likely to start FTPNC than multiparous women (χ^2^ = 6.8372, p = 0.0089). Women with accurate knowledge about FTPNC were more likely to obtain FTPNC (χ^2^ = 29.280, p < .001) and more likely to have seen a doctor within the past year (χ^2^ = 5.550, p = .018). Logistic regression confirmed that multiparity was associated with FTPNC and also that living in Texas was negatively associated with FTPNC (R^2^ = 0.066, F(9,340) = 2.662, p = .005). Among 27 women with non-FTPNC, barriers included late pregnancy recognition (n = 19) and no medical insurance (n = 5). *Conclusions* This study supports research that first time pregnancies have lower FTPNC, and demonstrated a strong association between delayed PNC and late pregnancy recognition. Strengthened investments in preconception planning could improve FTPNC in this population.

## Significance

This study found that multiparous Hispanic women have higher rates of FTPNC in U.S.-Mexico border communities than primiparous Hispanic women; age, country where care is accessed and Healthy Start participation were among the variables controlled for in the analyses. Programs aiming to improve FTPNC rates among Hispanic populations along the U.S.-Mexico border should target interventions to support women with preconception care and reproductive planning so that they identify pregnancies earlier. Improving FTPNC among women in the U.S.-Mexico border region may positively impact birth outcomes and the overall health status of women in this region.

## Introduction

First trimester prenatal care (FTPNC) is widely recognized to have numerous benefits for women and babies, and is associated with positive birth outcomes (Partridge et al. 2012; Taylor et al. 2005). Women accessing prenatal care (PNC) early on receive the full benefits of treating medical conditions, identifying and reducing potential risks, and addressing behavioral and environmental factors that contribute to poor outcomes.

The American Academy of Pediatrics and American College of Obstetricians and Gynecologists Guidelines for Perinatal Care (2012) recommends that women begin receiving prenatal care (PNC) in their first trimester of pregnancy. Healthy People 2020 has a goal of 77.6% of women obtaining FTPNC (DHHS 2013); and increasing FTPNC is one of four Healthy Border 2020 maternal and child health goals (USMBHC 2010).

Although federal efforts have had some success in increasing FTPNC in the general population, low-income and ethnic minority women continue to lag behind national rates (Luecken et al. 2009). Nationally and along the border, about 69% of Hispanic women had FTPNC in 2012, compared to 79% of white, non-Hispanic women (DHHS 2014). Hispanic women have greater maternal risk factors that can be positively impacted by early and adequate PNC, including higher rates of preterm birth than non-Hispanic white women (11.58 vs. 10.29%; DHHS 2014) and higher rates of neural tube defects (CDC 2010).

The higher prevalence of socioeconomic and demographic risk factors for preterm birth and other adverse outcomes among Hispanic women may be even more pronounced in the border region, where women have higher fertility rates (90.8 births per 1000 women vs. 86.5 for US women overall), higher rates of late or no prenatal care (14% compared to 7.7% among women in non-border regions) and higher rates of adolescent and teen pregnancy (13% higher among adolescents aged 15–19 and 20% higher among women aged 20–24 than non-border rates in these groups; McDonald et al. 2013); as well as higher rates of unintended pregnancies (48–62% across border states vs. 45% in the US; Kost 2010). Moreover, an estimated 73% of border counties are considered Medically Underserved Areas and 63% Health Professional Shortage Areas for primary medical care (National Rural Health Association 2010). In addition, the systemic and political issues surrounding immigration force many families with mixed immigration status into isolation and reluctance to engage with the health care system (Chavez et al. 2012).

Many studies have examined the sociodemographic and attitudinal factors that affect Hispanic women delaying PNC, but few look at how these may be different in the border context, a gap in knowledge which this study aims to inform. Factors associated with prenatal care-seeking among Hispanic women generally include: socioeconomic stressors (Luecken et al. 2009); work and transportation (Torres 2005); low education (Sunil et al. 2010); and negative experiences with providers that affect trust (Baxley and Ibitayo 2015). Along the border, factors with the potential to exacerbate these challenges include greater language barriers, fear and lack of social supports among large immigrant populations (Bergman and Connaughton 2013; Korinek and Smith 2011), challenges of prolonged and persistent poverty in border communities, high rates of uninsurance (Rosenberg et al. 2007), and an overstretched and under-resourced healthcare system in the region (National Rural Health Association 2010).

**Fig. 1:**
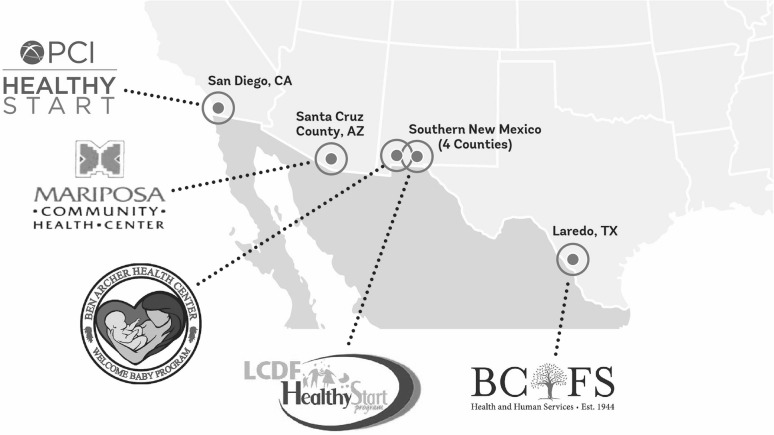
Map of Healthy Start Border Alliance sites

The Healthy Start Border Alliance (HSBA) is an alliance of five U.S.-Mexico border based Healthy Start program sites federally funded by the US Department of Health and Human Services [DHHS] Health Resources and Services Administration eliminate disparities in perinatal health. Healthy Start programs work to eliminate racial and ethnic disparities in perinatal health in areas with the highest poverty and poorest birth outcomes in their respective counties, by facilitating access to the health care system using culturally and linguistically tailored approaches proven to reduce barriers of fear and mistrust, in particular through home visits by trained community health worker/*Promotoras*. Along the border, Healthy Start is implemented by distinct agencies in San Diego County, California; Santa Cruz County, Arizona; Doña Ana, Luna, Otero and Sierra counties in New Mexico; and, Webb County, Texas. Formed in 2014, the HSBA is working to align its efforts to achieve measurable impact on maternal and child health among underserved border communities (Fig. 1).

### Objectives

This study aimed to identify (1) what demographic, knowledge and care-seeking factors influence first trimester prenatal care-seeking among pregnant and parenting Hispanic women in U.S.-Mexico border counties served by five Healthy Start sites, and (2) what barriers to FTPNC may be unique to this target population.

## Methods

This study was conducted with under the leadership of Project Concern International (PCI) in San Diego, CA. Institutional Review Board (IRB) review of the protocol resulted in exempt status for this research because of the use of retrospective data. All survey respondents provided informed consent in their native language.

The study targeted Hispanic women of reproductive age (15–44 years) residing along the U.S.-Mexico border in the area served by each Healthy Start implementing organization. Eligible survey respondents were identified based on a standardized protocol which required each site to stratify potential participants with a goal of at least 60% being non-Healthy Start participants and from outside of related clinic settings in order to minimize any program bias effect. Each site conducted outreach within its target catchment areas. Survey implementation sub-areas were identified by staff and outreach workers to parallel ongoing enrollment outreach strategies at each site that outreach women at a combination of venues, including door-to-door, street outreach, and community centers and events.

A 12-question survey was designed to assess key factors related to PNC seeking and possible barriers. Survey questions addressed demographic factors (age, race, parity); care-seeking practices (location of PNC; timing of last non-pregnancy related doctor visit; Healthy Start participation); knowledge and sources PNC information (clinical, friends and family, public information, community sources); and barriers to accessing FTPNC (issues related to insurance, clinical practices, fear or intimidation, familial or social support; logistical or scheduling challenges, late recognition of the pregnancy/unintended pregnancy, and perceived value of PNC). Late recognition of pregnancy (“I didn’t know I was pregnant until later”) and unintended pregnancy when given as reason for late PNC were combined into one category based on the assumption that these two factors often coincide.

Training and field testing across sites was conducted with a convenience sample of participants with non-FTPNC at three sites. Thereafter, the survey tool was refined, translated and adapted to a mobile app platform for data collection.

Across the five project sites, 536 women completed surveys administered by 19 Healthy Start case managers, outreach workers and other staff between November–December 2015, representing a 98.9% response rate. A total of 133 surveys were excluded as incomplete or not conforming to age, border residency or Hispanic ethnicity requirements. The data were analyzed using SAS (Version 9.3) and SPSS (Version 23). Chi square Tests of Independence were used to determine the association among various factors, including early PNC entry during the most recent pregnancy, Healthy Start participation, sources of information, knowledge, location of care, age, number of previous pregnancies, and timing of last doctor visit. A logistic regression model was included to determine the factors associated with FTPNC. For the logistic regression, an additional 47 surveys were excluded because of missing data on the dependent variable (first trimester PNC).

## Results

Of the 403 Hispanic survey respondents, most respondents were of White race (91%), and most had multiple pregnancies (70%; Table 1). The mean age was 29 years, with nearly 50% in the 25–34 age range. Almost 30% were current or past Healthy Start participants, most received PNC solely in the U.S. (86%), and most had a non-pregnancy related doctor visit within the last year. Approximately 14% of women accessed all or some of their PNC in Mexico, ranging from 29% of respondents in Arizona to 7–9% in New Mexico.

**Table 1:** Participant demographic and service data, by location

	San Diego County, CA	Santa Cruz County, AZ	Doña Ana County, NM	Four County Area, Southern NM	Webb County, TX	Overall
Respondents Total (n)	65	56	134	96	52	403
White race (%)	82	100	85	95	100	91
Mean age (years)	30.8	28.8	29.3	28.3	28.9	29.2
Healthy start participants (%)	26%	27	28	36	33	29
# Pregnancies (%)						
1 time	33	22	28	32	32	30
2–3 times	49	58	47	55	34	49
4+ times	18	20	25	13	34	21
Location of PNC (%)						
U.S. only	76	71	92	91	86	86
Mexico only	11	7	4	4	6	6
Both	11	22	5	3	8	8
Had a non-PNC-related doctor visit in the last year	83	91	87	90	63	84

Timing of PNC for the most recent pregnancy was split into two categories: first trimester and non-first trimester. The latter included those who began PNC in the 2nd or 3rd trimester as well as those who did not receive PNC. Of 353 women who had at least one pregnancy, a very high 92% received FTPNC (95% CI 89.1–94.9%; Table 2). Demographic and care-seeking factors that could potentially be associated with early PNC were examined, specifically: maternal age, number of pregnancies, location, Healthy Start participation, location of PNC and having a non-PNC doctor visit in the past year (Table 2). Chi square analyses revealed that women who were pregnant for the first time were significantly less likely to start FTPNC than women with multiple pregnancies (87 vs. 95%; χ^2^ (1, 353) = 6.8372, p = .0089).

**Table 2:** Comparison, by variable, of participants who had FTPNC vs. those who did not

Factor	# Respondents	1st trimester PNC (%)	Late or no PNC (%)
Overall	353	92.35	7.65
Age group (years)			
16–18	13	100	0
19–24	79	90	10
25–34	168	95	5
35–44	93	88	12
Number of pregnancies*			
One	105	87	13
Two or more	248	95	5
Healthy start participant			
Yes	118	95	5
No	235	91	9
Location of care			
US	304	93	7
Mexico	20	90	10
Both	29	90	10
Last saw doctor for health			
Past 6 months	206	94	6
Past 12 months	95	91	9
Over 1 year ago	52	90	10
Site			
CA	55	93	7
AZ	45	98	2
NM-1	110	93	7
NM-2	93	95	5
TX	50	82	16

*Significant variable: χ^2^ (1, 353) = 6.8372, p=0.0089. NM-1: Doña Ana County, New Mexico; NM-2: Four County Area, New Mexico

Knowledge about PNC, both in terms of number and sources of information on PNC and accuracy of knowledge about when to initiate PNC, were investigated in relationship to the same demographic and care-seeking factors as those by which FTPNC was analyzed. Women with accurate knowledge on when to start PNC were more likely to obtain FTPNC [χ^2^ (1, 353) = 29.280, p < .001] and were more likely to have seen a doctor for non-pregnancy related reasons within the past year [χ^2^ (1, 399) = 5.550, p = .018].

Across all sites, the top three most cited sources of information on when to start PNC were clinicians (64% of respondents), the WIC program (32%), and family and friends (24%). Although clinicians were the most important source of information on PNC, those who mentioned clinicians were not more likely to have more accurate information nor were they more likely to have obtained FTPNC.

While still high in our sample, FTPNC was lower among the youngest and oldest groups. FTPNC was slightly higher for those who had obtained PNC exclusively in the US (93 vs. 90%), and was lower the longer since their most recent non-pregnancy related doctor appointment, however none of these were significantly associated with higher FTPNC.

Although the differences were not significant, Healthy Start participants had higher rates of FTPNC (95 vs. 91%), lower rates of late or no PNC (5 vs. 9%), were less likely to have sought any PNC in Mexico (6 vs. 10%). In addition, a higher proportion of Healthy Start participants visited the doctor in the last six months for reasons other than PNC (65 vs. 54%) and reported more information sources on PNC, with 50% citing two or more information sources on PNC compared to 35% of non-Healthy Start participants (35%).

**Fig. 2:**
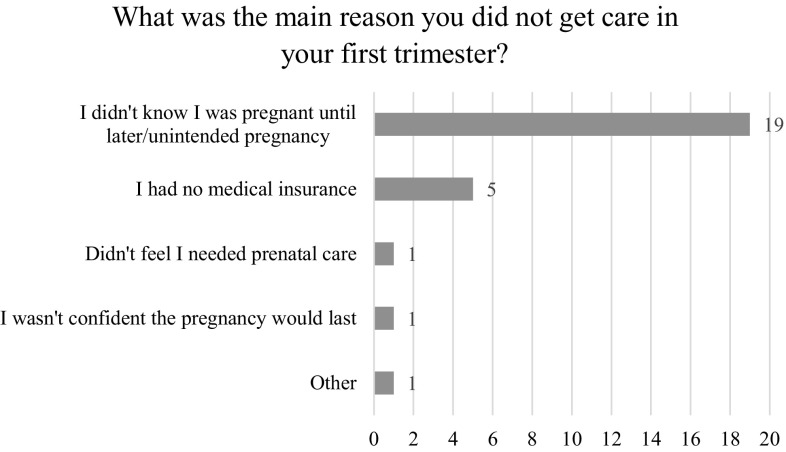
Most common reasons for late initiation of prenatal care

A total of 27 women were identified through the survey as having started PNC in the 2nd or 3rd trimester, or not at all. Across all sites, the most significant reason for women not receiving FTPNC was not knowing they were pregnant (n = 19; 70%) (Fig. 2). Five women (18.52%) across all sites indicated that they experienced barriers related to lack of insurance.

Among the 27 women who didn’t know they were pregnant, 53% had given accurate responses about when one should start PNC and 95% had been to the doctor in the last year for non-pregnancy related reasons. Consistent with the finding on parity, 68% of this subgroup were pregnant for the first time, compared to only 12% of those who mentioned other barriers. Because the sample of non FTPNC respondents was so small, it was not viable to run tests of significance on associations between barriers cited and other demographic or care-seeking practices.

Logistic regression was conducted to determine factors significantly associated with receiving FTPNC; demographic and service-related factors were included as covariates. Two variables explained approximately 6% of the variance (R^2^ = 0.066, F(9,340) = 2.662, p = .005). Having two or more pregnancies was associated with PNC in the first trimester (β= 0.167, p = .003). Living in Texas was negatively associated with having first trimester PNC (β = − .134, p = .029). Respondents in Texas were significantly less likely to have obtained FTPNC, with 82.4% obtaining FTPNC compared to 92–97% in other states.

## Discussion

The finding that primiparous women in our study were significantly less likely to obtain FTPNC contrasts with other studies that demonstrate higher parity as a risk factor for lower FTPNC among Hispanic women (Herbst et al. 2003; Meikle et al. 1995), and as a factor associated with inadequate PNC among migrant women (Heaman et al. 2013). Women who have been pregnant before may be more familiar with pregnancy symptoms, and have had recent experiences accessing PNC, making them more likely to know they are pregnant sooner and know how to enroll in PNC. This finding should be explored further to better understand how age and number of pregnancies interact with FTPNC among multiparous women.

The fact that Texas respondents were significantly less likely to have obtained FTPNC and that more Texas respondents mentioned lack of insurance as a barrier is reflective of the overall higher rates of uninsured in Texas (Barnett and Vornovitsky 2016). While the primary reported barrier to FTPNC in Texas was late pregnancy recognition, three of the five women in the study reporting lack of insurance as their primary barrier to FTPNC were in Texas. In the absence of more inclusive policies to expand health insurance in Texas, the role of Healthy Start and similar programs to link women and families to low cost services is even more critical.

Having accurate knowledge about when to start PNC was naturally associated with higher FTPNC. However, accurate knowledge about PNC was not associated with having received information from a clinician, despite the fact that most respondents mentioned clinicians as the primary source of information on PNC. Nearly a quarter of women in our study had not received information from any sources regarding when to start PNC, regardless of whether they had been to the doctor at any point. In particular, the youngest women in our sample had received the least amount of information on PNC, with one-third of 16–18 year olds having received no information on when to start PNC. As a trusted source of information among women in this study, clinicians can play a key proactive role in educating women on pregnancy risk, family planning and early signs of pregnancy, in particular among women who have not yet become pregnant. In addition, Healthy Starts and other programs have developed tools for reproductive life planning that have the potential to reduce unplanned pregnancies if they are incorporated routinely into well care for women of reproductive age and their partners.

It was expected that low rates of FTPNC in the border Healthy Starts and in the border in general would be associated with the barriers that Healthy Starts seek to overcome (including low insurance and lack of medical home; misunderstanding, fear or apprehension of the health care system; and linguistic and cultural concerns). While these barriers remain highly important, they were not significant for FTPNC in this sample. The top barrier to FTPNC identified by those survey respondents with non-FTPNC was simply that they did not know they were pregnant until after the first trimester. This is reflective of high rates of unintended pregnancy in the border region (Kost 2010), and is in line with other studies that point out late pregnancy discovery as a reason for delayed PNC and a key modifiable factor in improving FTPNC rates (Ayoola et al. 2010; Boerleider et al. 2015). This result suggest the need for strategies to improve early pregnancy recognition alongside efforts to reduce high rates of unintended pregnancies in the border region, if FTPNC in target areas is to be improved. Given the significant association between parity and FTPNC in our study, these efforts should focus in particular on nulliparous women.

Perhaps the most important finding in this study was that self-reported FTPNC rates assessed across all group were much higher than official FTPNC statistics in the target areas. This finding invites further exploration of how FTPNC is understood and reported among the target population. Unlike previous studies (Baxley and Ibitayo 2015; Bergman and Connaughton 2013), the vast majority of women in this sample knew the importance of PNC and provided an accurate response about when to initiate it. While the survey did not assess income and education level of respondents, the generally high rates of poverty and low educational attainment in the target areas render it improbable that the sample was uniquely socioeconomically advantaged, thereby explaining the high FTPNC rates assessed. Healthy Start participant respondents had significantly higher FTPNC than project data at each site (94.92% of Healthy Start survey respondents vs. 61.75% of Healthy Start project participants at all sites), perhaps suggesting that the recruitment methods somehow favored care-seekers, despite a careful sampling protocol. Since nearly 50% of the sample was multiparous and from the 25–34 age group with the highest FTPNC rate, controlling for parity and age in future studies may yield more useful information on disparities across subgroups.

Higher than expected FTPNC in this study may be influenced by the way FTPNC is locally reported in official data. As suggested by McDonald and colleagues (2015), PNC among Hispanic immigrants in the border region may be underreported, due to incomplete medical records, PNC received in Mexico which is not included, language difficulties and lack of confidence in women’s self-reports among birth certificate clerks. High rates of cross-border utilization of PNC in the sample, particularly in California (21.82%) and Arizona (28.89%) cannot alone explain the high rates of FTPNC assessed in the study. Nevertheless, this phenomenon should be examined more closely to ensure that public health data is accurately capturing information for women whose first PNC visit may have been in Mexico, and should include providers from border counties in Mexico to build a binational continuum of data sharing, alignment of definitions and standards. In fact, as pointed out by Lapeyrouse et al. (2012) and Byrd and Law (2009), access to another health care system offers border populations, particularly the poorer and less acculturated, access to alternative sources of care, and policies should be put in place to ensure that option is easy and safe.

Finally the possibility that Healthy Start projects are contributing broad public awareness of PNC and related supports in their target areas should not be ruled out as they promote successful approaches in the region.

### Study Limitations

Certain limitations to the study should be considered. The eligible sample size in each site is small and may not be representative of the respective target areas, and therefore may limit generalizability. Also, potentially important sociodemographic variables were also not assessed by the survey, e.g., education, income, and time in the U.S., nor was adequacy or continuity of PNC, which limits our ability to assess important confounding variables. The recruitment strategy at several sites may have favored respondents who were already connected with the health care system. In addition, we were limited in ability to detect factors significantly associated with FTPNC because of the small number of women who did not receive FTPNC in our sample. Thus only the factors most strongly related to FTPNC in our sample, were identified as significant. With a larger sample of women who did not receive FTPNC, we may have been able to identify more risk factors.

## Conclusions for Practice

Key findings from the study will inform Healthy Start Border Alliance strategies to improve FTPNC among this population and to inform discussions on how FTPNC is reported in the border region.

It is expected that this study will contribute to the evidence base around factors associated, and not associated, with FTPNC among Hispanic women living along the border. It is also hoped that this study will encourage additional research and inform policy and programs to address the challenges faced by women in the region.

Results of this study suggest that strengthened program investments in preconception care and reproductive planning, particularly among nulliparous women, can potentially make an important difference in women identifying pregnancies earlier and accessing important supports as early as possible, to ensure that every infant is given the best chance at a Healthy Start in life.
